# Native Entomopathogenic Fungi Isolated from *Rhynchophorus palmarum* (Linnaeus, 1758) in Northeast Brazil

**DOI:** 10.3390/insects15030159

**Published:** 2024-02-27

**Authors:** Viviane Araujo Dalbon, Juan Pablo Molina Acevedo, Karlos Antônio Lisboa Ribeiro Junior, João Manoel da Silva, Mayra Machado de Medeiros Ferro, Aldomário Santo Negrisoli Júnior, Henrique Goulart Fonseca, Antônio Euzébio Goulart Santana, Francesco Porcelli

**Affiliations:** 1 Laboratory of Natural Product Research, Campus for Engineering and Agrarian Sciences, Federal University of Alagoas, Maceió 57072-900, AL, Brazil; 2 Colombian Corporation for Agricultural Research C. I. Turipana, Montería 230002, Colombia; 3 Laboratory of Natural Products and Mass Spectrometry, Faculty of Pharmaceutical Sciences, Food and Nutrition, Federal University of Mato Grosso do Sul, Campo Grande 79070-900, MS, Brazil; karlos.lisboa@ufms.br; 4 Soil Laboratory, Federal Institute of Education, Science and Technology of Alagoas—IFAL, Santana do Ipanema Campus, Santana do Ipanema 57500-000, AL, Brazil; agrobio.jm@gmail.com; 5 Phytopathology Research Laboratory, Center for Engineering and Agrarian Sciences, Federal University of Alagoas, Rio Largo 57072-900, AL, Brazil; 6 Brazilian Agricultural Research Company—Embrapa Tabuleiros Costeiros, Aracaju 49025-040, SE, Brazil; 7 Dipartimento di Scienze del Suolo, della Pianta e degli Alimenti, University of Bari Aldo Moro, Campus Universitario Ernesto Quagliariello, Via Amendola, 165/A, 70126 Bari, Italy; francesco.porcelli@uniba.it

**Keywords:** genetic diversity, entomopathogenic fungi, biocontrol, invasive pest

## Abstract

**Simple Summary:**

Microbial pest control offers promising opportunities to develop sustainable alternatives to synthetic chemical insecticides. In Brazil, the selection of native strains of entomopathogenic fungi suggests an option for South American palm weevil (*Rhynchophorus palmarum*, SAPW) management and potential tools for red palm weevil (*R. ferrugineus*, RPW) management. Both weevils in America are susceptible to the native isolates of *Beauveria bassiana,* CVAD01, CVAD02, CVAD06, and CPATC/032, which originate from palm orchard soils and infected SAPW adults in northwestern Brazil’s Alagoas state. The SAPW mortality rates in our study were 90 and 100% on day 21, suggesting the efficacy of these strains that are well-adapted to the environment and to the pest. We suggest both the development of formulates for microbiological insecticides against *R. palmarum* and future tests on *R. ferrugineus.*

**Abstract:**

Both palm weevils, the South American (*Rhynchophorus palmarum*) (SAPW) and the red palm weevil (*R. ferrugineus*, RPW), are present in South America, affecting commercial, ornamental, and native palms. These pests oviposit and thrive on selected Arecaceae. *R. palmarum* mainly infests coconut (*Cocos nucifera*), oil palms (*Elaeis guineensis*), and other ornamental and native palms in America, causing a significant social impact on growers. The weevils fulfill a significant ectosymbiotic macro- and microorganism role in the first period of larval development, worsening the damage which, during this period, is not yet apparent. Palm protection in the Brazilian context suggests the use of indigenous agents for microbiological biocontrol. This research identifies three Brazilian *Beauveria bassiana* isolates: CVAD01, CVAD02, and CVAD06. The results suggest that the strain’s impact on *R. palmarum* can also be compared with that of the commercial strain *Beauveria bassiana*. Phylogenetic analysis allowed the delimitation of species of *Beauveria* (Hypocreales). Pathogenicity tests caused significant mortality in *R. palmarum*. The isolates CVAD01, CVAD02, and CVADO6 showed high pathogenicity between 7 and 21 days, with mortality rates between 90 and 100%, suggesting that they may be effective biological control agents of *R. palmarum* in the field when used, within available means, to mitigate the impact of *R. palmarum* and *R. ferrugineus* in South America.

## 1. Introduction

The coconut, *Cocos nucifera* (L.), and Arecaceae originate in central Malesia and the southwest Pacific [1] and have been spreading to the Neotropics by way of trade and cultivation. Coconut has now rapidly spread along the Brazilian northeastern coast region, as this region presents favorable edaphoclimatic characteristics for its cultivation; in addition, the ecosystem presents few possibilities for other commercial holdings. Thus, coconut holds a tremendous social significance for the area, constituting a basis for handcrafts and for the raw material needed for the cosmetics and food industry and finding use in the construction of houses. Fresh coconut’s use in traditional practices and its generation of employment and income opportunities for communities also constitute activities of social significance [2,3].

The incidence of insect pests and diseases is the main limitation on the production of coconut. Pests affect the plant from seedling to adult stages, affecting production [4]. *Rhynchophorus palmarum* (Linnaeus, 1758) (SAPW) causes significant losses to producers due to the larvae’s cryptic habit of tunnelling inside the palm stipe. SAPWs are difficult to control. South American palm weevil is the main pest in the region of Alagoas and is the vector responsible for the transmission of the nematode *Bursaphelencus cocophilus* (Cobb) Baujard (Parasitaphelenchidae), the causative agent of a red-ring-like disease in palms [5].

The geographical distribution of SAPW encompasses the American continent from Argentina to California and includes the Central American Antilles (EPPO, 2020). In addition, SAPWs have been reported in all of the Brazilian areas of commercial palm production. SAPW causes relevant economic damages in coconut plantations (*Cocos nucifera* L.) and oil palm (*Elaeis guineensis* Jacq.) plantations [6].

Adults SAPWs are black; a reddish atypical chromatic natural polymorphism can also be found [7]. Adults measure between 35 and 60 mm; their snouts demonstrate sexual dimorphism. The male snout is straight and stout, showing a brush-like series of seta on the fronto-clypeal region, while the female rostrum is slender, devoid of seta, and slightly dorso-ventrally arched [8].

Among the different techniques used to mitigate the damage caused by *R. palmarum,* synthetic chemical control is frequently applied but is less effective than needed for pest management. The traditional approach consists in uprooting and burning infested palms, which reduces infestation but results in intense environmental impact in the emission of greenhouse gases and waste management. Rhinchophorol aggregation pheromone, in combination with pieces of sugarcane, can help [9,10,11] in mass trapping programs.

Before applying a general pest management strategy for control, usually chemical control, an on-site survey should work to explore and identify new promising microbial agents [12]. Species of entomopathogenic fungi with significant potential for inclusion in bioinsecticides may already be living in the available environment [13,14,15]. For [16,17,18], native EF can serve as biopesticides with which to control insects of the genus *Rhynchophorus*, thus minimizing synthetic insecticide impact. The criteria for selecting isolates for biocontrol purposes are the insect mortality rates observed in bioassays and efficient conidia production in culture medium [19,20].

Different techniques can help identify EF; among these, the morphological taxonomic technique facilitates the scrutiny of pathogenicity tests [21]. Later, molecular approaches resulted in more precise strain identification for industrial mass production of a native EF-specific strain. The advancement of molecular techniques, especially those based on DNA analysis by polymerase chain reaction (PCR), has allowed the development of fast, accurate, and applicable methodologies for many samples, allowing the detection and identification of different fungi [22,23]. In this sense, DNA profiles have been used as powerful and sensitive tools with which to accurately identify fungal isolates that infect a host population [24]. Molecular techniques are helpful tools in the study of the phylogenetic identification of fungi, being necessary for the sequencing of several specific regions for species identification [23,24].

Among the molecular markers, the internal transcript spacer (ITS) region is commonly used for the phylogeny of entomopathogenic fungi. Nuclear markers have highly conserved sequences [23] and translation elongation factor-1α (α-TEF) can aid in differentiation at the level of species [23]. Therefore, the present study aimed to characterize the genetic diversity of native entomopathogenic fungi that infect *R. palmarum* and verify the pathogenicity of these isolates to *R. palmarum,* suggesting that they may also serve to control *R. ferrugineus*.

## 2. Material and Methods

### 2.1. Study Site

The four sampling sites in this study were coconut plantations at Sao José Farm in the town of Coruripe, Alagoas. Point “A” is located at 10°08′30.5″ S, 36°12′07.4″ W; this location is named “hybrid coconuts” or “giant coconuts” and comprises 5 hectares, producing 3000 fruits per month. Areas “B” (10°08′26.4″ S, 36°12′08.3″ W) and “C” (10°08′36.0″ S, 36°12′05.6″ W), with green dwarf coconuts on 7 hectares each, produce 7000 to 8000 green fruit per month. All of the plantations were exclusively dedicated to the commercial production of coconut water. A total of 100 samples of insects with symptoms of fungal infection underwent the sampling methodology [13] and were incubated in moist chambers at 28 ± 1 °C to promote fungal development [21]. The isolates were classified as CVAD01, CVADO2, and CVAD06, according to the areas in which they were collected (Suplementary Figure S1; Table 1).

### 2.2. Morphological Characterization of Native Entomopathogenic Fungi

Morphological identification was carried out during microculture fungi growth [25,26]. Thalli macroscopic analysis considered primary isolation growth speed, recto and verso color, mycelium topography, and texture. Microscopic identification was carried out with a Zeiss Stemi 305 CAM digital stereo zoom microscope and photomicrography, verifying the presence of septa, conidiophores, and phialides, the fungal structures used for taxonomic identification of native EF isolates. A fungal key [27] was used for insect pathogen identification. 

### 2.3. Isolation of Entomopathogenic Fungi from Rhynchophorus palmarum

The three fungal isolates from SAPWs in Coruripe-Al, Brazil, had been previously identified as CVAD01, CVAD02, and CVAD06. *Beauveria bassiana* CPATC/032 (Embrapa, Coastal Tablelands) and a commercial isolate of *B. bassiana*^®^ served for control for the bioassays of pathogenicity and mortality analysis (Table 1).

The EF from *R. palmarum*—CVAD01, CVAD02, and CVAD06—were isolated from infected weevils by surface-sterilizing the insect-invaded fungal area with surface-sterilizing agents (HgCl_2_ (0.1%)/NaOCl (2%)). Then, to isolate the strains, the isolates were grown on the vegetative structures of the fungus on a sequence of PDA medium (potato dextrose agar) in Petri dishes at 25 °C for 20 days. After growth and sporulation, the isolates were used to reinfect the *R. palmarum* adult to verify Koch’s postulates. Then, we isolated the pathogen, multiplied it, and identified the organism responsible for the disease (the isolate was purified three different times) [28]. 

### 2.4. Insect Pathogenicity Bioassay

A sterile scalpel collected fungal propagules from the Petri dishes for suspension in sterile water with 5 mL/L of Tween 80 and test tube transfer. A Neubauer chamber with a double mirror allowed spore counting up to 10^9^ spores/mL. *R. palmarum* adults were soaked in 20 mL for two cycles of three seconds each. The same technique, without conidia, served for the control. After soaking, each weevil was placed in a sterile plastic flask with a filter paper floor and sugarcane food chips. All of the flask-related items were tested via separate plating on nutrient agar, a basic culture medium commonly used for the culture of entomopathogenic fungi, to ensure that they were fungus-free. Then, flasks were scrutinized on a daily basis until the weevil’s death. Soon after death, we washed and rinsed each weevil, first in 70% (*v*/*v*) EtOH and then in sterile water. Subsequently, the weevils were isolated in moist chambers. We waited for fungal growth to develop, confirming the cause of death of the weevil. Dead insects were moved daily, with a distilled water-moistened cotton swab, to sterile plastic boxes and placed into a thermo-refrigerated cabinet (TRC) with temperature, humidity, and photoperiod control. The experiments were conducted in air-conditioned rooms and in (TCR) 5 ± 2 °C, 70 ± 10%, and 12 h of photophase.

During the experiment, at intervals of 7, 14, 21, and 28 days, the (%) mortality of the treatments was calculated and corrected for the control according to the formula established by Shneider-Orelli (1947): M (%) = ((M − Mcontrol)/(100 − Mcontrol)) × 100.

### 2.5. DNA Extraction and PCR Amplification

Seven-day-old PDA cultures at room temperature offered mycelia for DNA extraction and amplification, following the modified protocol [29]. The crushed mycelium were placed in 1.5 mL microcentrifuge tubes. Then, 1 mL of extraction buffer hexadecyltrimethylammonium bromide (CTAB) 4% (CTAB, 4%; NaCl, 1.4 M; EDTA, 20 mM; Tris-HCl, 100 mM; PVP, 1%) and 4 μL of β-mercaptoethanol (0.1% *v*/*v*) were added. The mixture rested for 30 min at 65 °C in a water bath. Then, the mixture was centrifuged for 15 min at 12,000 rpm. The supernatant was placed in a new tube and then diluted with 600 μL of ASD (chloroform: isoamyl alcohol 24:1) and 40 μL of 10% CTAB heated to 65 °C. After centrifugation, the aqueous phase was placed in a new tube with 400 μL of absolute EtOH. DNA was precipitated, washed with 70% EtOH, and dried at room temperature before resuspension in 40 μL of TE (Tris-EDTA: Tris-HCl, 10 mM; EDTA, 1 mM) + RNAse (10 μg/mL). Agarose gel (0.8%), ethidium bromide, and UV light allowed visual estimation of DNA quality of the mixture stored at −20 °C.

PCR amplification of the ITS region was conducted using the primer sets ITS1 forward (5′TCCGTAGGTGAACCTGCGG3′), ITS4 (5′TCCTCCGCTTATTGATATGC3′), EFl-n8F (5′CATCGAGAAGTTCGAGAAGG3′), and EFl-986R (5′TACTTGAAGGAACCCTTACC3′). A total volume of 30 μL was processed. The final reaction volume obtained, as measured with autoclaved Milli-Q water, was 60 μL. An Applied Biosystems thermocycler (2720 Thermal Cycler) ran the following PCR reactions for ITS: the protocol described below was used for 1 cycle at 94 °C for 2 min (initial denaturation), 35 cycles at 94 °C for 45 s (denaturing), 55 °C for 30 s (annealing), 72 °C for 35 s (extension), and, finally, for 1 cycle at 72 °C for 10 min [29]. For TEF, the following conditions were applied: 95 °C for 8 min, 35 cycles at 95 °C for 15 s, 55 °C for 20 s, 72 °C for 60 s, and, finally, 1 cycle at 72 °C for 5 min [30]. Electrophoresis was then applied to the amplified mixture in 1.5% agarose gel. Then, ethidium bromide staining and UV light observation (Macrogen Inc., Seoul, Republic of Korea) were used to sequence the amplified PCR products.

### 2.6. Data Editing and Phylogenetic Analysis

The nucleotide sequences were aligned with the CodonCode Aligner version 11.0 software (CodonCode Corporation, Dedham, MA, USA), and sense and antisense sequences comparison analysis was used to correct nucleotide positions in ambiguous sequences. The BLASTn algorithm [31] produced the initial analyses in the GenBank database https://www.ncbi.nlm.nih.gov/genbank/ (Last accessed on 26 December 2023), the same repository offered reference sequences, used for the phylogenetic tree reconstruction, for several species of the genus (Supplementary Table S1).

MEGA X [32] was used to obtain multiple alignments for the nucleotide sequences. CIPRES portal, using MrBayes v.3.2.3 [32], was utilized to construct individual Bayesian inference (BI) phylogenies for the ITS and TEF sequences. Concatenated sequences obtained by MrModeltest v.2.3 constituted the best evolutionary model for the analyses, according to the Akaike information criterion (AIC) [33]. The analyses were carried out over 10 million generations using four chains and sampled every 1000 generations for 10,000 trees. The first 2500 trees were discarded in the burning phase. Then, probabilities [34] were determined from a majority rule consensus tree generated with the remaining 7500 trees.

### 2.7. Statistical Analysis

Pathogenicity assay was applied according to [35] in a fully randomized experimental design with 6 treatments and 12 replicates; each repetition included an adult insect. ANOVA testing and Duncan mean comparison were carried out; in both cases *p* ≤ 0.05 indicated significance. During the experiment, we calculated the mortality (%) of the treatments at 7-, 14-, 21-, and 28-day intervals, correcting these calculations with the control.

## 3. Results and Discussion

Initially, we purified the fungi isolates according to Koch’s postulates [35]. However, after three different bioassays, we used the calculated size of the inoculum to confirm the efficiency of any isolates for the pest’s cause of death. If not replicated rapidly, the entomopathogenic fungi and the native isolates lose their virulence after purification. In this study, the virulence was maintained at a high level in all of the bioassays. This allowed us to confirm that the pathogen was an effective tool, demonstrating a high virulence and naturally causing a high mortality rate in the pest.

### 3.1. Morphophysiological Characterization of Native Entomopathogenic Fungi

**Figure 1:**
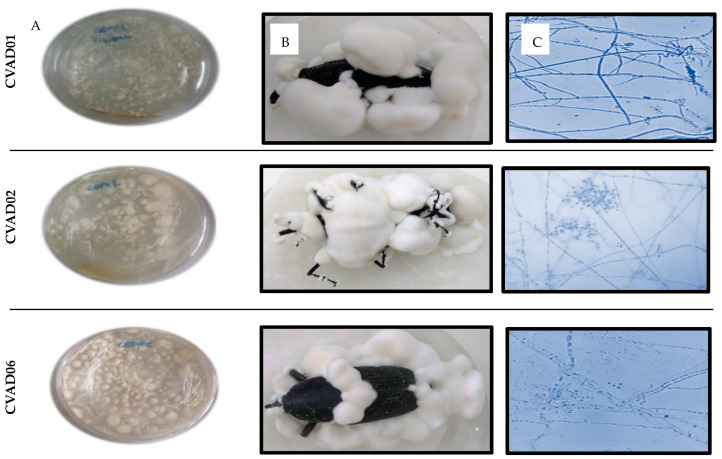
Photographs of colonies in PDA medium (bar = 1 cm) (**A**). Sporulation of *B. bassiana* in adult *R. palmarum* with 14 days of infection (**B**). Spores and hyphal structures of *Beauveria* isolates observed with compound microscope (400× magnification) (**C**).

The three native isolates (CVAD01, CVAD02, and CVAD06) and the controls (CPATC/032 and Commercial) show the same macroscopic characteristics: slow growth, white mycelium, a light-yellow reverse topography of the cottony mycelium, and powdery texture (Figure 1A). Morphological scrutiny helped characterize the entomopathogenic fungi in our study, facilitating taxonomic identification and driving the selection of isolates used in pathogenicity tests [14]. Nevertheless, we relied on the work of [36], using only fungal isolated material for morphological species identification, thus preventing morphological changes due to repeated steps on artificial media and waiting for confirmation of molecular analysis identification. The microscopic analyses of the isolates returned photomicrographs. The hyphae were hyaline septate with uniform septa, geniculate conidiophores, zigzag denticulae, single-celled agglomerated phialides, and ovoid-shaped conidia (Figure 1B). All isolates presented morphological characteristics consistent with the genus *Beauveria* as reported by [37,38] and showed spore germination (Figure 1C), indicating that the isolates were of a good quality. In agreement with observations by [38], we also observed a wide variation between strains of the genus *Beauveria* spp. [39].

The genus *Beauveria* is a cosmopolitan entity with anamorphic and teleomorph stages. It is found in soil and insects and demonstrates facultative and highly entomopathogenic characteristics. *Beauveria* causes significant pathogenicity in arthropods; as such, insect pest management has been applied to it [27,36,40]. Species identification in *Beauveria* is difficult due to the genus’ structural simplicity and lack of characteristics in phenotypic variation [41].

In general, infection with *Beauveria* begins with adhesion. Subsequently, conidia germinate on the host’s cuticle. This is followed by enzyme production. Appressorium differentiation completes the hyphae’s entrance into the insect body. The process culminates with the death of the host and differentiation and dispersal of new conidia [42].

Therefore, this study highlights the importance of morphological studies for entomopathogenic fungi, prioritizing bioassays to determine mortality, pathogenicity, and fungi grouping, as molecular analyses require a longer time to be completed. Thus, this study may be of great importance for the identification of the species.

### 3.2. Evaluation of Mortality and Pathogenicity of Native Entomopathogenic Fungi in R. palmarum

Isolates from native EF-infected *R. palmarum* were available from August to December 2018. A study of isolates demonstrated that EF infection occurs naturally in the county of Coruripe, Alagoas, Brazil. According to [14], the species of entomopathogenic fungi with the greatest potential for use as a bioinsecticide are those that are cosmopolitan; that is, they may be found in the environment in which the microorganism will be applied.

Study of the five *B. bassiana* isolates, CVAD01/CVAD02/CVAD06, and of the controls *B. bassiana* CPATC/032 and *B. bassiana* Commercial, revealed spore germination, suggesting the good quality of the isolates and their suitability for pathogenicity tests. According to [43], germination quality is directly related to the viability of conidia and the survival time of the isolates. During the bioassays, the EF infection grew on the insect, covering the insect and sporulating, as described by [14,43]. *Beauveria bassiana* isolates CVAD01/CVAD02/CVAD06, *B. bassiana* CPATC/032, and *B. bassiana* Commercial completed their cycle on *R. palmarum* in 19, 20, 21, 23, and 25 days, respectively. BOD was carried out at 25 ± 2 °C with an RH of 70 ± 10% and 12 h of photophase. Sporulation development began in the spiracles, in the membranes of segments, in the legs, in the bases of antennas, and on the rostrum of *R. palmarum*. Our observations correspond with those recorded in [42,43] while analyzing the pathogenicity of promising isolates of *B. bassiana* on adults of *R. palmarum*.

Evaluating the corrected mortality rates (Table 2) of *R. palmarum* that derived from the five isolates, CVAD01, CVAD02, CVAD06, Commercial, CPATC/32, and native *B. bassiana*, we noted that, during the first seven days, CVAD06 reached a 50% corrected mortality rate, a statistically significant difference (*p* ≤ 0.05) compared with the other isolates (*B. bassiana*-isolated CVAD01, *B. bassiana* Commercial, and *Beauveria* sp. CVAD02).

Within 14 days, the isolates *B. bassiana*-isolated CVAD06 and *B. bassiana*-isolated CVAD01 produced 70% mortality; within 21 days, the corrected mortality rates were 100% across all isolates of *B. bassiana*, including CVAD06, CVAD01, and CPATC/32. These results demonstrate a statistically significant difference (*p* ≤ 0.05) compared with *B. bassiana* Commercial, which demonstrated a 70% corrected mortality rate.

Finally, on day 28, the native isolates maintained a 100% corrected mortality rate, and the corrected mortality rate of *B. bassiana* Commercial increased to 91%. These results primarily demonstrate that the mortality rates of the native isolates, from the 14th day of the evaluation to the 21st day, rose from 70 to 100%, providing evidence of their promising potential for the biological control of *R. palmarum* adults. The high mortality rate obtained demonstrates that fungi isolates have adapted to eliminate the growth and multiplication of *R. palmarum* adults in the northeastern region of Brazil and indicates that these pathogens have coevolved with the pest, adapting in order to survive in this environment. The authors of [43,44,45] suggest that *B. bassiana* constitutes a widespread biological control agent against a wide range of insect pests worldwide and that *B. bassiana* are associated with and adapted to different habitat types, insect hosts, and specific environmental conditions.

In Table 3, a second experiment under the same bioassay conditions and with previous multiplication of all isolates in *R. palmarum* adults (to reactivate virulence) showed that, at day 7, the native isolate *Beauveria* sp. CVAD02 inflicted a 50% corrected mortality rate, different (*p* ≤ 0.05) from the isolated CVAD01, isolated CVAD06, Commercial, and CPATC/32. At day 14, CVAD01 demonstrated a 100% corrected mortality rate, followed by CVAD02 and CVAD06, which both demonstrated a 91.6% mortality rate, significantly different (*p* ≤ 0.05) from the mortality rates produced by CPATC/32 and Commercial, which were 45% and 50%, respectively. When evaluated at day 21, mortality rates differed for CVAD06 and Commercial, with mortality rates increasing to 58% and 54%, respectively. No further increase in mortality rate was observed at 28 days. Thus, the native CVAD01, CVAD02, and CVAD06 showed high pathogenicity against adult *R. palmarum* infection.

Table 4 shows data comparable to the previous similar experiments, compiled after reactivating the isolates’ pathogenicity on *R. palmarum*. Seven days after inoculation, the isolate CVAD06 induced a mortality rate that was 50% different (*p* ≤ 0.05) from other isolates. At day 14, CVAD01 produced a 100% mortality rate and CVAD02 produced a 91% mortality rate; these results are significantly different (*p* ≤ 0.05) from those of the commercial fungi CVAD06 (72%) and CPATC/32 (75%). On day 21, the mortality rate of the two native isolates and CPATC/32 increased to 91.6%.

### 3.3. Molecular Identification of Native Entomopathogenic Fungi

In this study, the native entomopathogenic fungi presented good quality, integrity, purity, and suitability for amplification. Approximately 544 bases were determined for ITS and 329 bases for TEF; congruence analyses revealed no conflict between the datasets. The maximum likelihood (ML) and Bayesian inference (BI) trees that were generated presented the same topology; therefore, only the BI tree was presented in this study. The combined dataset of the two loci included 30 taxa with *Cordyceps tenuipes* (ARSEF 4096) as external group taxa, facilitating the rooting of the phylogenetic tree and the temporal organization of the analyzed species. The concatenated alignment had 1511 characters, of which 141 were parsimonious informational sites, 1083 were conserved sites, and 428 were variable sites. The locus limits in the alignments were as follows: 1-906 for ITS and 907-1511 for TEF. For phylogenetic analyses of BI, according to AIC, the GTR + I + G model was selected for ITS and SYM + G was selected for TEF; for the combined data, the model used GTR + I + G.

**Figure 2:**
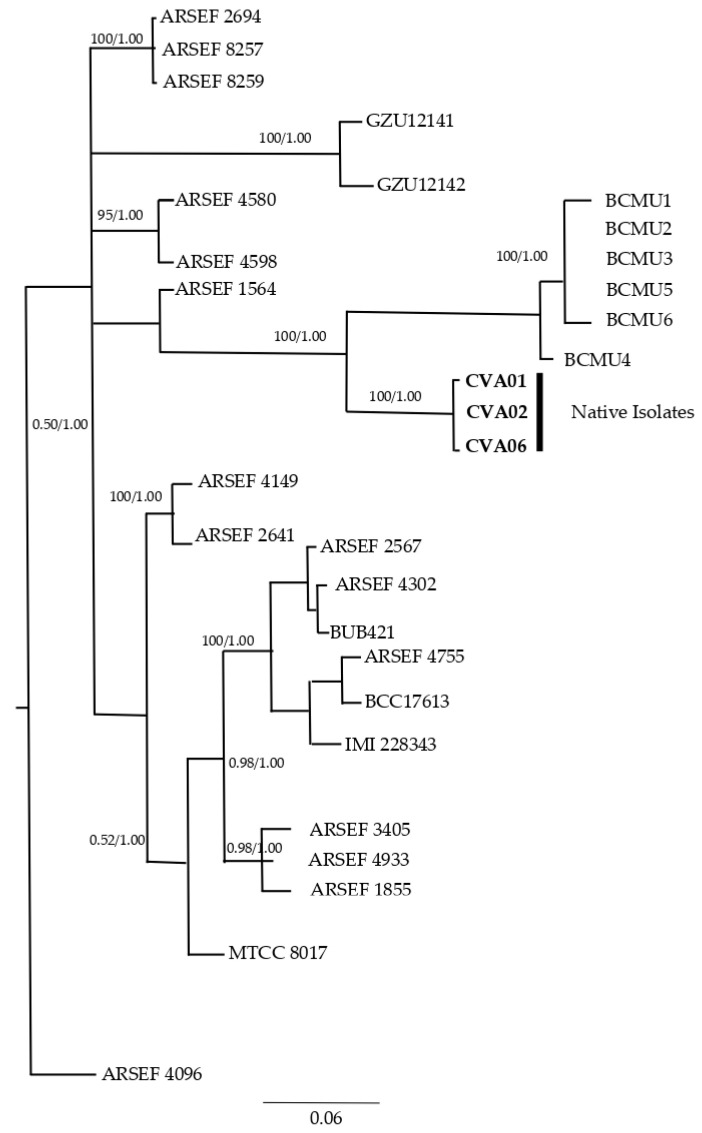
Phylogenetic tree of Bayesian inference of *Beauveria bassiana* isolates based on concatenated sequences of ITS and TEF genes. Bayesian posterior probability values > 0.55 are indicated on the nodes. *Cordycepes tenuipes* was used as an outside group. The isolates obtained in this study are represented by CVAD1, CVAD2, and CVD6. The scale bar (0.06) represents nucleotide substitutions per site. The analyses took place over 10 million generations, using 4 chains, and were sampled every 1000 generations, for a total of 10,000 trees.

Phylogenetic analyses for the ITS and TEF regions allowed the delimitation of the *Beauveria bassiana* (Hypocreales) species (Figure 2; Supplementary Figures S2 and S3) the same regions were used by the authors of [46] to identify naturally occurring entomopathogenic fungi isolated from banana soil as biocontrol agents of *Cosmopolites sordidus* (Germar, 1824; Coleoptera: Curculionidae).

For the authors of [47], it was important to use different gene regions when identifying the species in the genus *Beauveria* that have simple structures, thus requiring robust identification at the species level. This morphological fungal relationship is also supported by [47], suggesting that species identification in *Beauveria* is difficult due to their structural simplicity and lack of distinct phenotypic variation, in turn highlighting the importance of multilocus sequence studies when establishing species boundaries in *Beauveria*; however, for this study, the regions analyzed allowed the identification of isolates for *B. bassiana*.

When amplifying the DNA extracted [48] from the native fungi from parasitized *R. ferrugineus*, the amplification fragments showed 600 bp and homology, with 100% similarity, for *B. bassiana*, with reference sequences deposited in GenBank. The results from [47] are like those obtained in this study: indigenous entomopathogenic fungal isolates showed 100% sequence similarity with *B. bassiana,* as the three analyzed strains were taxonomically grouped. The data presented here and obtained by repeated bioassays suggest that the geographic origin and pathogenicity of native EF isolates possess an excellent mix of controls to manage the target insect, *R. palmarum*.

A better understanding of the *R. palmarum* population structure in the Coruripe, Alagoas, coconut tree crop area will help to evaluate the ecological and potential impact of the biocontrol action of EF. According to [48] the species of entomopathogenic fungi with the greatest potential to be developed as bioinsecticides are those that are cosmopolitan and found in the environment in which the microorganism will be applied. The authors of [49,50] have shown that native microbial agents can be used in biological control programs for *Rhynchophorus* spp. populations.

For the authors of [50], entomopathogenic fungi, such as *B. bassiana*, *Metarhizium anisopliae*, *Isaria fumosorosea*, and *Lecanicillium lecanii*, are a promising alternative for the management of *R. ferrugineus* infestations when used with other control methods, such as pheromone traps, insecticides, and sanitation, significantly reducing RPW populations and damage.

Meanwhile, the exotic species of entomopathogenic fungi used in biocontrol may be ineffective in some pests due to their adaptation to climatic diversity and host-related isolate differences; however, the identification of native EF is considered a promising alternative due to their ecological suitability to native pest species and the lower non-target organisms’ susceptibility when compared with exotic isolates [49,50].

## 4. Conclusions

According to the findings of this research study, the native Brazilian isolates of *Beauveria bassiana* (CVAD2, CVAD1, and CVD6) identified in this study caused high pathogenicity and mortality for *R. palmarum* and were found to be promising for biocontrol in palms. The commercial isolate achieved lower mortality rates in the management of *R. palmarum* and in the potential control of *R. ferrugineus* in Brazil. However, field studies are needed to reach sound conclusions and achieve practical applications.

Classical morphological analysis allowed the identification of the fungi’s native Brazilian isolates up to the level of the genus *Beauveria* spp. However, it is necessary to carry out phylogenetic analysis in the ITS and TEF regions to delimit and define this species from *Beauveria bassiana* (Hypocreales).

## Figures and Tables

**Table 1:** Names and origins of the entomopathogenic fungal isolates used in this study.

Isolate Name	Insect Species	Source of Isolation	Origin	Geographic Location
CVAD01	*R. palmarum*	Adult	Coruripe, Alagoas, Brazil	10°08′30.5″ S 36°12′07.4″ W
CVAD02	*R. palmarum*	Adult	Coruripe, Alagoas, Brazil	10°08′26.4″ S 36°12′08.3″ W
CVAD06	*R. palmarum*	Adult	Coruripe, Alagoas, Brazil	10°08′36.0″ S 36°12′05.6″ W
*B. bassiana* CPATC/032	*R. palmarum*	Adult	Embrapa Tabuleiros Costeiros, SE, Brazil	Not applicable
*B. bassiana* (^®^)	Not applicable	Not applicable	Not applicable	Not applicable

**Table 2:** Mortality percentage of native EF on *R. palmarum*. PM—percentage of mortality; PMC—percentage of corrected mortality; x—Mean; SD—standard deviation.

Day	Experiment 1									
	*B. bassiana* CVAD01		*B. bassiana* CVAD02		*B. bassiana* CVAD06		*B. bassiana* CPATC/32		*B. bassiana* (^®^)	
	PM	PMC	PM	PMC	PM	PMC	PM	PMC	PM	PMC
	x	x ± SD	x	x ± SD	x	x ± SD	x	x ± SD	x	x ± SD
7	25	10 ± 3.768 b	25	10 ± 3.768 b	50	40 ± 4.351 a	8.3	0	25	10 ± 3.768 b
14	75	70 ± 5.179 a	58.3	50 ± 4.291 c	75	70 ± 3.768 a	66.6	60 ± 4.103 b	66.6	60 ± 4.103 b
21	100	100 ± 3.553 a	100	100 ± 1.855 a	100	100 ± 1.855 a	100	100 ± 1.855 a	75	70 ± 3.768 b
28	100	100 ± 3.553 a	100	100 ± 1.855 a	100	100 ± 1.855 a	100	100 ± 1.855 a	91	90 ± 2.405 b

Mean with the same letter are not significantly different (Duncan *p* ≤ 0.05).

**Table 3:** Mortality percentage of native EF on *R. palmarum*. PM—percentage of mortality; PMC—percentage of corrected mortality; x—Mean; SD—standard deviation.

Day	Experiment 2									
	*B. bassiana* CVAD01		*B. bassiana* CVAD02		*B. bassiana* CVAD06		*B. bassiana* CPATC/32		*B. bassiana* (^®^)	
	PM	PMC	PM	PMC	PM	PMC	PM	PMC	PM	PMC
	x	x ± SD	x	x ± SD	x	x ± SD	x	x ± SD	x	x ± SD
7	33.3	3.3 ± 4.103 b	16.6	16.6 ± 3.243 d	50	50 ± 4.351 a	41.6	41.6 ± 4.291 ab	41.6	41.6 ± 4.291 ab
14	100	100 ± 1.86 a	91.6	91.6 ± 2.405 b	75	72.7 ± 3.768 c	75	75 ± 3.768 c	75	75 ± 3.768 c
21	100	100 ± 1.86 a	91.6	91.6 ± 2.405 b	75	75 ± 3.768 c	91.6	91.6 ± 2.405 b	75	75 ± 3.768 c
28	100	100 ± 1.86 a	91.6	91.6 ± 2.405 b	75	75 ± 3.768 c	91.6	91.6 ± 2.405 b	75	75 ± 3.768 c

Mean with the same letter are not significantly different (Duncan *p* ≤ 0.05).

**Table 4:** Mortality percentage of native EF on *R. palmarum*. PM—percentage of mortality; PMC—percentage of corrected mortality; x—Mean; SD—standard deviation.

Day	Experiment 3									
	*B. bassiana* CVAD01		*B. bassiana* CVAD02		*B. bassiana* CVAD06		*B. bassiana* CPATC/32		*B. bassiana* (^®^)	
	PMC	PM	PMC	PM	PMC	PMC	PM	PMC	PM	PMC
	x	x ± SD	x	x ± SD	x	x ± SD	x	x ± SD	x	x ± SD
7	33	3.3 ± 4.103 b	50	50 ± 4.351 a	25	25 ± 3.768 c	25	25 ± 3.768 c	25	25 ± 3.76 c
14	100	100 ± 1.86 a	91.6	91.6 ± 2.405 b	91.6	91.6 ± 2.405 b	50	50 ± 4.351 c	50	45.4 ± 2.51 d
21	100	100 ± 1.86 a	91.6	91.6 ± 2.405 b	91.6	91.6 ± 2.405 b	58.3	58.3 ± 4.291 cd	58.3	54.5 ± 4.291 b
28	100	100 ± 1.86 a	91.6	91.6 ± 2.405 b	91.6	91.6 ± 2.405 b	66.6	63.6 ± 4.103 d	83	81.8 ± 3.243 c

Mean with the same letter are not significantly different (Duncan *p* ≤ 0.05).

## Data Availability

All relevant data are within the paper.

## Supplementary Materials

The following supporting information can be downloaded at: https://www.mdpi.com/article/10.3390/insects15030159/s1. Supplementary Figure S1: Geographical location of sampling area; Supplementary Figure S2: Phylogenetic tree of isolates of *Beauveria bassiana* isolates through Bayesian analysis of the ITS intergenic region. Supplementary Figure S3: Phylogenetic tree of isolates of *Beauveria bassiana* isolates through Bayesian analysis of the TEF intergenic region. Supplementary Table S1: Taxa and their GenBank accession numbers used in the phylogenetic analysis.

## References

[1] POWO—Plants of the World Online (2023). Facilitated by the Royal Botanic Gardens, Kew. https://powo.science.kew.org/taxon/urn:lsid:ipni.org:names:666160-1.

[2] Elias G.A., Santos R. (2016). Produtos Florestais Não Madeireiros e Valor Potencial de Exploração Sustentável da Floresta Atlântica no sul de Santa Catarina. Ciência Florest..

[3] Elias G.A., Santos R., Citadini-Zanette V., Corrêa P. (2015). Arecaceae: Bibliometric analysis of the native species of the state of Santa Catarina, Brazil. Ciência Nat..

[4] Ferreira J.M.S., Warwick D.R.N., Siqueira L.A. (2018). A Cultura do Coqueiro no Brasil.

[5] Moens M., Perry R.N. (2009). Migratory Plant Endoparasitic Nematodes: A Group Rich in Contrasts and Divergence. Annu. Rev. Phytopathol..

[6] Dalbon V.A., Acevedo J.P.M., Ribeiro Junior K.A.L., Ribeiro T.F.L., da Silva J.M., Fonseca H.G., Santana A.E.G., Porcelli F. (2021). Perspectives for Synergic Blends of Attractive Sources in South American Palm Weevil Mass Trapping: Waiting for the Red Palm Weevil Brazil Invasion. Insects.

[7] Löhr B., Vásquez-Ordóñez A.A., BecerraLopez-Lavalle L.A. (2015). *Rhynchophorus palmarum* in Disguise: Undescribed Polymorphism in the“Black” Palm Weevil. PLoS ONE.

[8] Gallo D., Nakano O., Silveira Neto S., Carvalho R.P.L., de Baptista G.C., Berti filho E., Parra J.R., Zucchi R.A., Alves S.B., Vendramim J.D. (2002). Entomologia Agricola.

[9] Ferreira J.M.S. (2002). Coco, Fitossanidade.

[10] Duarte A.G., De Lima I.S., Ferraz D.M.A., Santana A.E.G. (2003). Captura de *Rhynchophorus palmarum* L. (Coleóptera: Curculionidae) em armadilhas iscadas con o feromônio de agregação e compostos voláteis de frutos do abacaxi. Rev. Bras. Frutic..

[11] Moura J.I.L., Resende M.L.V., Sgrillo R., Nascimento L.A., Romano R. (1990). Diferente tipos de armadilhas de iscas no controle de *Rhynchophorus palmarum* L. (Coleóptera: Curculionidae). J. Agrotrópica.

[12] Rochat D., Malosse C., Lettere M., Ducrot P.H., Zagatti P., Renou M.D. (1991). Male-produced aggregation pheromone of the American palm weevil, *Rhynchophorus palmarum* (L.) (Coleóptera: Curculionidae), collection, identification, electrophysiological activity and laboratory bioassay. J. Chem. Ecol..

[13] Sanjuan T., Tabima J., Restrepo S., Laessoe T., Spatafora J.W., Franco-Molano A.E. (2014). Entomopathogens of Amazonian stick insects and locusts are members of the *Beauveria* species complex (*Cordyceps* sensu stricto). Mycologia.

[14] Alves S.B. (1998). Quantificação de inóculo de patógenos de inseto. Controle Microbiano de Insetos.

[15] Goettel M.S., Laird M., Lacey L.A., Davidson W. (1990). Safety to nontarget invertebrates of fungal biocontrol agents. Safety of Microbial Insecticides.

[16] Ahmed R., Freed S. (2021). Virulence of *Beauveria bassiana* Balsamo to red palm weevil, *Rhynchophorus ferrugineus* (Olivier) (Coleoptera: Curculionidae). Egypt. J. Biol. Pest Control.

[17] Wakil W., Yasin M., Shapiro-Ilan D. (2017). Effects of single and combined applications of entomopathogenic fungi and nematodes against *Rhynchophorus ferrugineus* (Olivier). Sci. Rep..

[18] Yasin M. (2017). Virulence of entomopathogenic fungi *Beauveria bassiana* and *Metarhizium anisopliae* against red palm weevil, *Rhynchophorus ferrugineus* (Olivier). Entomol. Res..

[19] Shahina F., Salma J., Mehreen G., Bhatti M.I., Tabassum K.A. (2009). Rearing of *Rhynchophorus ferrugineus* in laboratory and field conditions for carrying out various efficacy studies using EPNs. Pak. J. Nematol..

[20] McKinnon A.C., Glare T.R., Ridgway H.J., Mendoza-Mendoza A., Holyoake A., Godsoe W.K., Bufford J.L. (2018). Detection of the Entomopathogenic Fungus *Beauveria bassiana* in the Rhizosphere of Wound-Stressed *Zea mays* Plants. Front. Microbiol..

[21] Teixeira R.A., Faria M. (2010). Pequeno Manual Sobre Fungos Entomopatogênicos.

[22] Wang C., Wang S. (2017). Insect Pathogenic Fungi: Genomics, Molecular Interactions, and Genetic Improvements. Annu. Rev. Entomol..

[23] Imoulan A., Hussain M., Kirk P.M., Meziane A.E., Yao Y. (2017). Entomopathogenic fungus Beauveria: Host specificity, ecology and significance of morpho-molecular characterization in accurate taxonomic classification. J. Asia-Pac. Entomol..

[24] Schoch C.L., Seifert K.A., Huhndorf S., Robert V., Spouge J.L., Levesque C.A., Chen W., Bolchacova E., Fungal Barcoding Consortium, Fungal Barcoding Consortium Author List (2012). Nuclear ribosomal internal transcribed spacer (ITS) region as a universal barcode marker for Fungi. Proc. Natl. Acad. Sci. USA.

[25] Humber R. (2005). Entomopathogenic Fungal Identification.

[26] Koneman E., Winn W., Allen S., Janda W., Procop G., Schreckenberber P., Woods G. (2012). Koneman’s Colour Atlas and Textbook of Diagnostic Microbiology.

[27] De Hoog G.S. (1972). The genera *Beauveria*, *Isaria*, *Tritirachium*, *Paecylomices* and *Acrodontium*. Study Mycol..

[28] Michereff S.J. (2012). Fundamentos de Fitopatologia.

[29] Dellaporta S.L., Wood J., Hicks J.B. (1983). A plant DNA minipreparation: Version II. Plant Mol. Biol. Report..

[30] Castlebury L.A., Rossman A.Y., Sung G.H., Hyten A.S., Spatafora J.W. (2004). Multigene phylogeny reveals new lineage for *Stachybotrys chartarum*, the indoor air fungus. Mycol. Res..

[31] Altschul S.F., Gish W., Miller W., Myers E.W., Lipman D.J. (1990). Basic local alignment search tool. J. Mol. Biol..

[32] Kumar S., Stecher G., Tamura K. (2016). MEGA7: Molecular Evolutionary Genetics Analysis Version 7.0 for Bigger Datasets. Mol. Biol. Evol..

[33] Posada D., Buckley T.R. (2004). Model selection and model averaging in phylogenetics: Advantages of akaike information criterion and bayesian approaches over likelihood ratio tests. Syst. Biol..

[34] Rannala B., Yang Z. (1996). Probability distribution of molecular evolutionary trees: A new method of phylogenetic inference. J. Mol. Evol..

[35] Bhunjun C.S., Phillips A.J., Jayawardena R.S., Promputtha I., Hyde K.D. (2021). Importance of molecular data to identify fungal plant pathogens and guidelines for pathogenicity testing based on Koch’s Postulates. Pathogens.

[36] Bustamante D.E., Oliva M., Leiva S., Mendoza J.E., Bobadilla L., Angulo G., Calderon M.S. (2019). Phylogeny and species delimitations in the entomopathogenic genus *Beauveria* (Hypocreales, Ascomycota), including the description of *B. peruviensis* sp. nov. MycoKeys.

[37] Varela A., Morales E. (1996). Characterization of Some *Beauveria bassiana* Isolates and Their Virulence toward the Coffee Berry Borer *Hypothenemus hampei*. J. Invertebr. Pathol..

[38] Ullah M.I., Arshad M., Abdullah A. (2018). Use of the entomopathogenic fungi *Beauveria bassiana* (Hyphomycetes: Moniliales) and *Isaria fumosorosea* (Hypocreales: Cordycipitaceae) to control *Diaphorina citri* Kuwayama (Hemiptera: Liviidae) under laboratory and semi-field conditions. Egypt. J. Biol. Pest Control.

[39] White T.J., Bruns T., Lee S., Taylor J. (1990). Amplification and Direct Sequencing of Fungal Ribosomal RNA Genes for Phylogenetics. PCR Protoc..

[40] Rehner S.A., Minnis A.M., Sung G.H., Luangsa-ard J.J., Devotto L., Humber R.A. (2011). Phylogeny and systematics of the anamorphic, entomopathogenic genus *Beauveria*. Mycologia.

[41] Picciotti U., Araujo Dalbon V., Ciancio A., Colagiero M., Cozzi G., De Bellis L., Finetti-Sialer M.M., Greco D., Ippolito A., Lahbib N. (2023). “Ectomosphere”: Insects and Microorganism Interactions. Microorganisms.

[42] Castrillo L.A., Wraight S.P., Galaini-Wraight S., Matsumoto T.K., Howes R.L., Keith L.M. (2020). Genetic diversity among naturally-occurring strains of *Beauveria bassiana* associated with the introduced coffee berry borer, *Hypothenemus hampei*, (Coleoptera: Curculionidae) on Hawai’i Island. J. Invertebr. Pathol..

[43] Gebremariam A., Chekol Y., Assefa F. (2021). Phenotypic, molecular, and virulence characterization of entomopathogenic fungi, *Beauveria bassiana* (Balsam) Vuillemin, and *Metarhizium anisopliae* (Metschn.) Sorokin from soil samples of Ethiopia for the development of mycoinsecticide. Heliyon.

[44] Leon-Martinez G.A. (2019). Patogenicidad y autodiseminación de cepas promisorias de hongos entomopatógenos sobre *Rhynchophorus palmarum* L. (Coleoptera: Dryophthoridae). Agron. Mesoam..

[45] Serna-Domínguez M.G., Andrade-Michel G.Y., Rosas-Valdez R., Castro-Félix P., Arredondo-Bernal H.C., Gallou A. (2019). High genetic diversity of the entomopathogenic fungus *Beauveria bassiana* in Colima, Mexico. J. Invertebr. Pathol..

[46] Lozano-Soria A., Picciotti U., Lopez-Moya F., Lopez-Cepero J., Porcelli F., Lopez-Llorca L.V. (2020). Volatile Organic Compounds from Entomopathogenic and Nematophagous Fungi, Repel Banana Black Weevil (*Cosmopolites sordidus*). Insects.

[47] Rehner S.A., Buckley E.P. (2005). 2005. A *Beauveria* phylogeny inferred from ITS and EF1-α sequences: Evidence for cryptic diversification and links to *Cordyceps teleomorphs*. Mycologia.

[48] Ment D., Levy N., Allouche A., Davidovitz M., Yaacobi G. (2023). Efficacy of Entomopathogenic Fungi as Prevention against Early Life Stages of the Red Palm Weevil, *Rhynchophorus ferrugineus* (Coleoptera: Curculionidae) in Laboratory and Greenhouse Trials. Insects.

[49] Pu Y.C., Zheng Z.W., Ding C.H. (2023). Development of potential microbial agents with two new entomopathogenic fungal strains to control the red palm weevil *Rhynchophorus ferrugineus* (Olivier) (Coleoptera: Curculionidae). Egypt. J. Biol. Pest Control.

[50] Sabbahi R., Hock V. (2024). Entomopathogenic fungi against the red palm weevil: Lab and field evidence. Crop Prot..

