# Supplementary material for: Native Entomopathogenic Fungi Isolated from Rhynchophorus palmarum (Linnaeus, 1758) in Northeast Brazil
Source: Insects. 2024 Feb 27;15(3):159. doi: 10.3390/insects15030159 (PMC10971377; doi:10.3390/insects15030159)
Supplement: Supplementary file 1 [file insects-15-00159-s001.zip › insects-2722233-supplementary.pdf]

Supplementary material

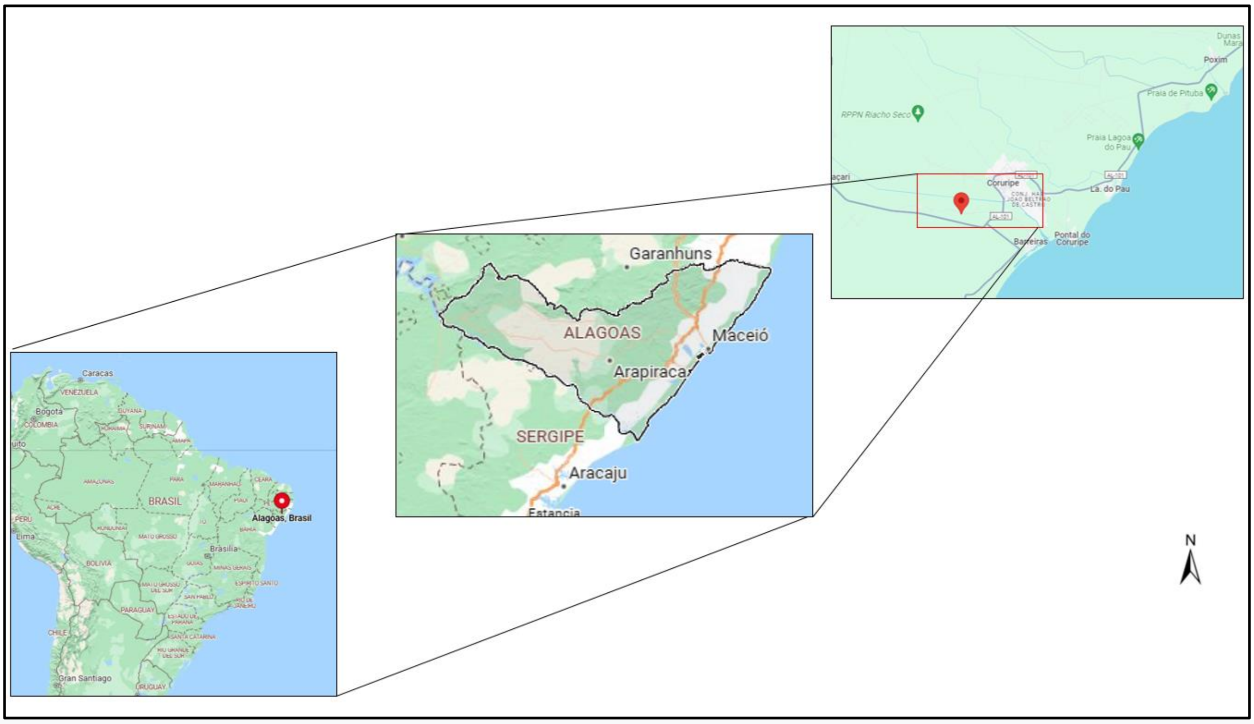

Supplementary Figure S1. Geographical location of sampling area (lower box) Brazil and the sampling sites in the state of Alagoas in Coruripe, (upper box).

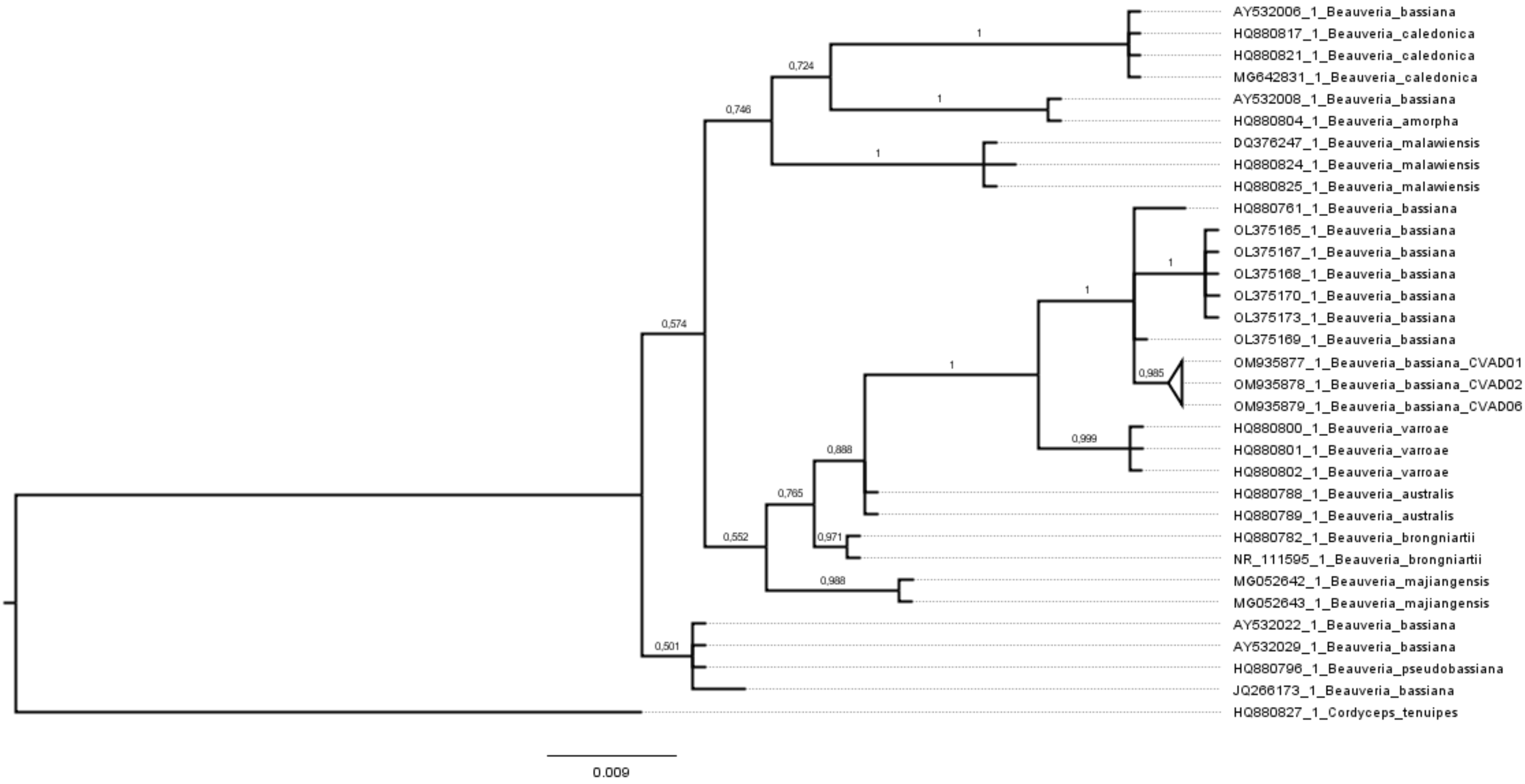

Supplementary Figura S2. Phylogenetic tree of isolates of *Beauveria bassiana* isolates through Bayesian analysis of the ITS intergenic region. Bayesian posterior probability values >0.55 are indicated on the nodes. *Isaria tenuipes* and *Isaria farinosa* was used as an outside group. The isolates obtained in this study are represented by CVAD2, CVAD1 and CVD6. The scale bar (0.009) represents nucleotide substitutions per site. The analyses took place over 10 million generations using four chains and sampled every 1,000 generations, for a total of 10,000 trees.

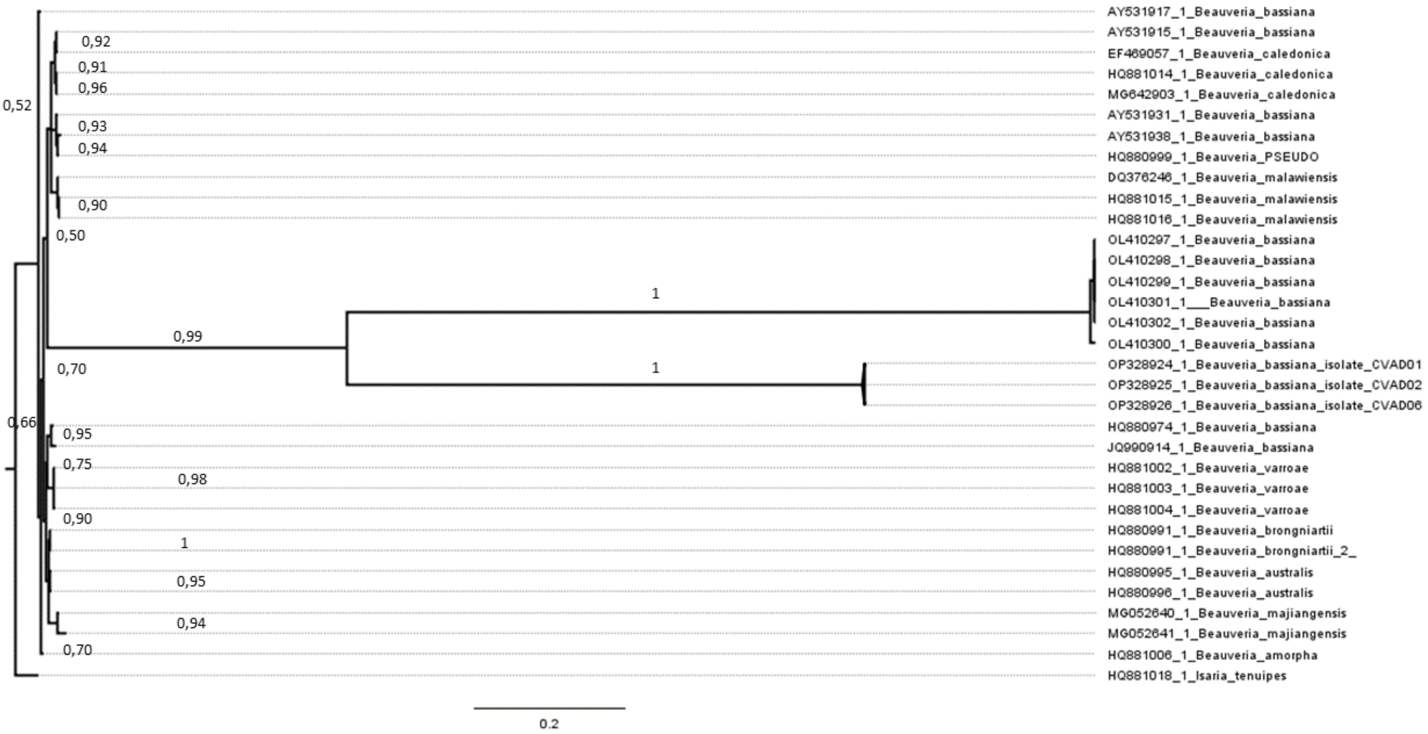

Supplementary Figure S3. Phylogenetic tree of isolates of *Beauveria bassiana* isolates through Bayesian analysis of the TEF intergenic region. Bayesian posterior probability values >0.82 are indicated at the nodes. *Metarhizium anisopliae* was used as an outgroup. The isolates obtained in this study are represented by CVAD2, CVAD1 and CVD6. Scale bar (0.02) represents nucleotide substitutions per site. Analyzes took place over 10 million generations using four strands and sampled every 1,000 generations, for a total of 10,000 trees. using MrBayes v. 3.2.3.

Supplementary Table S1. Taxa and their GenBank accession numbers used in the phylogenetic analysis.

Isolate Code	Species	Location	Host	GenBank Accession Numbers	
				ITS	TEF
**ARSEF 2567**	*B. amorpha*	Scotland	Soil	AY532006	AY531915
**ARSEF 2641**	*B. amorpha*	Brazil	Hymenoptera: Formicidae	AY532008	AY531917
**ARSEF 3405**	*B. bassiana*	USA	Lepidoptera: Lymantriidae	AY532022	AY531931
**ARSEF 4933**	*B. asiatica*	France	Coleoptera: Scolytidae	AY532029	AY531938
**IMI 228343**	*B. malawiensis*	USA	Clavicipitaceae, Hypocreales	DQ376247	DQ376246
**ARSEF 1564**	*B. bassiana*	Italy	Lepidoptera: Arctiidae	HQ880761	HQ880974
**ARSEF 4580**	*B. australis*	Australia	Orthoptera: Acridiidae	HQ880788	HQ880994
**ARSEF 4598**	*B. australis*	Australia	Soil	HQ880789	HQ880995
**ARSEF 1855**	*B. pseudobassiana*	Canada	Coleoptera: Scolytidae	HQ880796	HQ880999
**ARSEF 8257**	*B. varroae*	France	Acari: Varroidae	HQ880800	HQ881002
**ARSEF 8259**	*B. varroae*	France	Acari: Varroidae	HQ880801	HQ881003
**ARSEF 2694**	*B. varroae*	Switzerland	Coleoptera: Curculionidae	HQ880802	HQ881004
**ARSEF 4149**	*B. amorpha*	Australia	Coleoptera: Scarabaeidae	HQ880804	HQ881006
**ARSEF 4302**	*B. caledonica*	Switzerland	Soil	HQ880821	HQ881014
**BCC17613**	*B. malawiensis*	Australia	_	HQ880824	HQ881016
**ARSEF 4755**	*B. malawiensis*	Australia	Soil	HQ880825	HQ881015
**MTCC_8017**	*B. bassiana*	India	Lepidoptera: bombycidae	JQ266173	JQ990914
**GZU12141**	*B. majiangensis*	China	Coleoptera: Scarabaeoidea	MG052642	MG052640
**GZU12142**	*B. majiangensis*	China	Coleoptera: Scarabaeoidea	MG052643	MG052641
**BUB421**	*B. majiangensis*	China	Coleoptera: Scarabaeoidea	MG642831	MG642903
**CVAD 01***	*B. bassiana*	Brazil: Alagoas	Coleoptera: Curculionidae	OM935877	OP328924
**CVAD 02***	*B. bassiana*	Brazil: Alagoas	Coleoptera: Curculionidae	OM935878	OP328925
**CVAD 06***	*B. bassiana*	Brazil: Alagoas	Coleoptera: Curculionidae	OM935879	OP328926
**BCMU1**	*B. bassiana*	Thailand	Diptera: Tephritidae	OL375165	OL410297
**BCMU2**	*B. bassiana*	Thailand	Diptera: Tephritidae	OL375167	OL410298
**BCMU3**	*B. bassiana*	Thailand	Diptera: Tephritidae	OL375168	OL410299
**BCMU4**	*B. bassiana*	Thailand	Diptera: Tephritidae	OL375169	OL410300
**BCMU5**	*B. bassiana*	Thailand	Diptera: Tephritidae	OL375170	OL410301
**BCMU6**	*B. bassiana*	Thailand	Diptera: Tephritidae	OL375173	OL410302
			**Outgroup**		
**ARSEF 4096**	Cordyceps tenuipes	Brazil	Lepidoptera: Noctuidae	HQ880827	HQ881018
